# Performance of rGO/TiO_2_ Photocatalytic Membranes for Hydrogen Production

**DOI:** 10.3390/membranes10090218

**Published:** 2020-09-01

**Authors:** Juan Corredor, Eduardo Perez-Peña, Maria J. Rivero, Inmaculada Ortiz

**Affiliations:** Department of Chemical and Biomolecular Engineering, ETSIIT, University of Cantabria, Avda. de los Castros s/n, 39005 Santander, Spain; corredorj@unican.es (J.C.); eduardo.perezp@alumnos.unican.es (E.P.-P.); mariajose.rivero@unican.es (M.J.R.)

**Keywords:** rGO/TiO_2_, Nafion, hydrogen production, photocatalysis, photocatalytic membrane

## Abstract

Although there are promising environmental and energy characteristics for the photocatalytic production of hydrogen, two main drawbacks must be overcome before the large- scale deployment of the technology becomes a reality, (i) the low efficiency reported by state of the art photocatalysts and, (ii) the short life time and difficult recovery of the photocatalyst, issues that need research and development for new high performance catalysts. In this work 2% rGO/TiO_2_ composite photocatalysts were supported over Nafion membranes and the performance of the photocatalytic membrane was tested for hydrogen production from a 20% vol. methanol solution. Immobilization of the composite on Nafion membranes followed three different simple methods which preserve the photocatalyst structure: solvent-casting (SC), spraying (SP), and dip-coating (DP). The photocatalyst was included in the matrix membrane using the SC method, while it was located on the membrane surface in the SP and DP membranes showing less mass transfer limitations. The performance of the synthesized photocatalytic membranes for hydrogen production under UVA light irradiation was compared. Leaching of the catalytic membranes was tested by measuring the turbidity of the solution. With respect to catalyst leaching, both the SC and SP membranes provided very good results, the leaching being lower with the SC membrane. The best results in terms of initial hydrogen production rate (HPR) were obtained with the SP and DP membrane. The SP was selected as the most suitable method for photocatalytic hydrogen production due to the high HPR and the negligible photocatalyst leaching. Moreover, the stability of this membrane was studied for longer operation times. This work helps to improve the knowledge on the application of photocatalytic membranes for hydrogen production and contributes in facilitating the large-scale application of this process.

## 1. Introduction

In the current global energy framework and according to the new commitments to reduce greenhouse gas emissions (GHG), the use of renewable hydrogen is an interesting option to facilitate energy transition [1,2,3,4]. Nowadays, 95% of hydrogen is produced by steam reforming from fossil fuels, mainly natural gas, which is energy intensive and still contributes to GHG emissions [5,6,7]. The rest of the hydrogen is mainly produced by electrolysis, that today is particularly energy and cost intensive [8,9,10]. Other processes, less extensive, make use of renewable energy sources in the generation of hydrogen, e.g., biological processes, electrolysis combined with solar panels and photocatalysis [11,12] thus driven by solar light. The latter being an interesting alternative in terms of energy as it takes advantage of the use of waste effluents as sacrificial agents that contribute further to the transition from a linear to a circular economy.

State of the art heterogeneous photocatalysts have been widely investigated such as, metal oxides (TiO_2_, Cu_2_O), carbonaceous materials (g-C_3_N_4_), and chalcogenides (CdS, ZnS) [13]. Among the studied photocatalysts, TiO_2_ reports good properties such as chemical and thermal stability, high photo-reactivity, and low cost. However, its wide band gap (3.2 eV) restricts its applications to ultraviolet light, which represents only about 4–8% of the solar spectrum. An additional drawback is the high recombination rate of the electron-hole pairs, which reduces its photocatalytic activity [14,15]. Photocatalytic hydrogen production has been mainly studied using alcohols (such as methanol or ethanol) as sacrificial agents or performing only water splitting. Most of these studies used Pt coupled to the photocatalyst because of the higher hydrogen production achieved with this noble metal. This fact is due to the high value of the Pt work function (5.93 eV) which allows an efficient transfer of the photogenerated electrons from the photocatalyst to the noble metal [16]. However, Pt is a very scarce and high cost material [17,18]. Coupling TiO_2_ with graphene oxide (GO) results in a low-cost alternative to improve the photocatalytic activity of TiO_2_. GO is a two- dimensional planar sheet composed of sp^2^ bonded carbon atoms structured in a hexagonal lattice network highly functionalized with oxygenated groups. It can be reduced to graphene, which can improve the photocatalytic activity of TiO_2_ due to its ability to carry charges from the TiO_2_ conduction band reducing the electron-hole recombination rate. Moreover, the band gap shift can contribute by taking advantage of the solar spectrum [19,20].

Regarding the photocatalytic reactor configuration, slurry type reactors have been widely used in order to reduce mass transfer limitations. However, two major drawbacks are associated with the use of suspended solid reactors: (i) the requirement of a separation and recovery step of the photocatalyst after the photocatalytic process which is especially difficult with nanomaterials due to their small size and, (ii) hazards associated with the manipulation of nanomaterials [21]. Therefore, techniques to immobilize the photocatalyst leading to immobilized photocatalytic membrane reactors (IPMR) have been encouraged to facilitate catalyst reuse and to avoid the presence of nanomaterials in the treated waters and effluents. When using a membrane, it can offer a role of support to the photocatalyst and it can also act as selective barrier for the compounds to be degraded. In IPMRs where the membrane exerts an immobilization and filtration function, dead-end or cross- flow configurations are possible [22,23]. In the case of hydrogen production, most of the works use an H-type reactor which consists of two compartments, the first one containing the photoanode, the second one containing a Pt cathode and a proton exchange membrane that divides both compartments to allow H^+^ transport between both chambers. These reactors have the advantage of producing H_2_ and O_2_ separately. Most of the H-type reactors immobilize TiO_2_-based photocatalysts on a Ti foil and use Nafion as proton exchange membrane [24,25,26,27,28,29]. A cross-flow type reactor was employed by Hattori et al. They used a TiO_2_ nanotube array deposited on a Pd thin film to produce hydrogen from the reforming of low molecular weight alcohols. The generated hydrogen was purified through the Pd film [30]. With regard to hydrogen production with immobilized catalyst in a simple single chamber photoreactor using a membrane with the sole function of photocatalyst support only a few works can be found in the literature [31,32,33,34]. Some works have made use of glass substrates to support the photocatalyst; Cha et al. evaluated the effect of Pt location and amount in TiO_2_ nanotubes supported on a fluorine doped tin oxide (FTO) glass, using a methanol solution as sacrificial agent [31]. Ma et al. synthesized Er^3+^:YAlO_3_Pt-TiO_2_ composite on a glass substrate for hydrogen production from an aqueous solution of glucose [32]. Della Foglia immobilized Pt/TiO_2_ on glass fibers for photo-steam reforming of low molecular weight alcohols [33]. Wu et al. obtained hydrogen from an ethanol solution using immobilized TiO_2_/Pt and TiO_2_/Pd on cellulose membranes [35].

Photocatalysts have also been immobilized on polymeric membranes [35,36,37,38,39]. However, most of the polymer materials were damaged by UV irradiation. Polytetrafluoroethylene (PTFE) has been used as photocatalyst support because of its photochemical resistance [38,39]. Sulfonated polytetrafluoroethylene (Nafion) is resistant to photochemical degradation and in addition is a proton (H^+^) conductor due to the sulfonic acid groups attached to the PTFE backbone [40,41,42]. Nafion has been used as membrane support of the photocatalyst in the photocatalytic degradation of different pollutants [43,44,45]; it has been also employed as photocatalyst coating to improve the degradation of cationic molecules due to the anionic character of the sulfonic groups contained in Nafion membranes [46,47]. Regarding photocatalytic hydrogen production, Nafion has been used as a matrix to attach photocatalysts with visible light photosensitizers [34,48] as well as a proton exchange membrane in H-type reactors. Park et al. used (Ru(bpy)_3_^2+^) as photosensitizer, (methyl viologen) as electron mediator and Pt supported on a Nafion membrane for hydrogen production from ethylenediaminetetraacetic acid (EDTA) [34]. Choi bonded Ru(bpy)_3_^2+^ to TiO_2_ within a Nafion layer to produce hydrogen from an EDTA solution [48].

A wide variety of immobilization methods have been used in photocatalytic pollutant degradation, such as electrophoresis, chemical and physical vapor deposition, sol-gel, thermal spraying, and solvent deposition [49]. A reliable technique for photocatalyst immobilization must provide a strong photocatalyst support, uniform coating, high degree of photocatalyst irradiation, and preserve photocatalyst structure during preparation and immobilization [50]. In this work, two solvent deposition methods (SP and DP) and SC were compared because these methods are simple and the soft operational conditions do not provoke changes in the composite structure [49]. SC was chosen because it prevents photocatalyst leaching as it is embedded in the polymeric membrane. However, mass transfer limitations are expected to be higher than in the other methods. Lower mass transfer limitations are expected in DP and SP methods due to the location of the photocatalyst on the membrane surface; although DP is a simpler method, the SP method allows a uniform coating.

Although there is interesting information already reported in the literature, several gaps need to be filled before the large scale application of photocatalytic hydrogen production, such as: (i) the comparison of the performance of slurry type and IPMR for similar catalysts and operating conditions, and (ii) increasing photocatalyst stability and life time for long operation times. This study advances the knowledge of IPMRs by experimentally assessing the performance of newly synthesized rGO/TiO_2_ composite photocatalysts supported on Nafion polymeric membranes. Furthermore, several methods to immobilize the catalyst for photocatalytic hydrogen production in a one-chamber photoreactor were developed and the results compared in terms of hydrogen generation rate, membrane reuse capacity, and catalyst leaching.

## 2. Materials and Methods

### 2.1. Materials

Methanol HPLC grade was purchased from Scharlau and isopropanol 99.5% was supplied by Acros Organics (Madrid, Spain). TiO_2_ P25 was purchased from Evonik; a dispersion of graphene oxide sheets in water solvent and 4 mg mL^−1^ GO was provided by Graphenea; while 20% Nafion in alcohol solution and Nafion membranes N115, with a thickness of 127 µm according to the supplier, were purchased from Ion Power. Pure argon 3X from Praxair (Camargo, Spain) was used to ensure an inert atmosphere in the reactor.

### 2.2. Photocatalyst Preparation and Characterization

**Figure 1:**
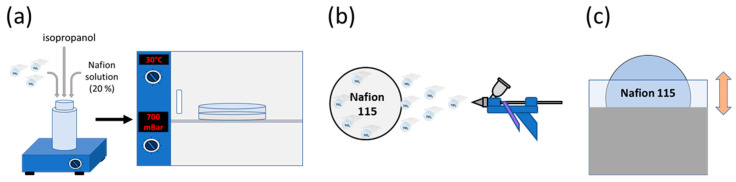
Photocatalytic immobilization methods: solvent-casting (SC) (**a**), spraying (SP) (**b**) and dip- coating (DP) (**c**).

The photocatalytic membrane diameter was 4.4 cm. The membranes were loaded with 10% *w*/*w* of photocatalyst. The photocatalyst was immobilized on Nafion membranes by three different methods: SC, SP, and DP. 2% (*w*/*w*) of rGO/TiO_2_ was hydrothermally synthesized as described in previous works [15,20]. Figure 1 illustrates the different immobilization methods.

In the SC method, 42.2 mg of photocatalyst were added to 1.81 g of Nafion solution and 0.5 mL of isopropanol. The mixture was stirred for 10 min and placed in an ultrasonication bath (Fisher scientific FB1505, Madrid, Spain) with a frequency of 37 kHz for 30 min. The resulting suspension was dried in a Petri dish set in a vacuum oven at 800 mbar and 30 °C over 24 h.

The SP method was applied to attach the composite catalyst to the Nafion N115 membrane. An ink composed of 0.3% photocatalyst, 2.7% of 5% Nafion solution, and 97.0% of isopropanol was prepared and ultrasonicated for 45 min at 37 kHz before being applied to the membrane. The membrane was placed on a heating plate at 60 °C while it was sprayed to achieve a photocatalyst concentration of 10% **w*/*w** on the membrane. After spraying, the membrane was dried for 24 h at ambient conditions.

The DP method was carried out by immersing the membrane for 10 min in a solution composed of 3.0% of photocatalyst, 25.7% of 5% Nafion solution, and 71.3% of isopropanol. The solution was dispersed using an ultrasonication bath for 45 min before it was applied. After each immersion, the membrane was dried at ambient conditions for 10 min. The membranes were immersed for 6 consecutive times to reach the desired concentration of photocatalyst. After the last immersion, the membrane was dried for 24 h at ambient conditions.

The detailed characterization of the rGO/TiO_2_ composite material can be found in previous works [13,15]. Membrane characterization was carried out through different techniques. Fourier transform infrared (FTIR) spectra were recorded on a Spectrum Two spectrometer (Perkin Elmer, Madrid, Spain) equipped with an attenuated total reflection (ATR) accessory. Thermogravimetric analysis (TGA) was carried out in a Shimadzu DTG-60H Differential Thermal Gravimetric Analyzer (Barcelona, Spain) by heating the samples under nitrogen atmosphere (50 mL min^−1^) from 25 °C to 900 °C at 10 °C min^−1^. Scanning electron microscopy images were recorded with a SEM EVO MA 15, Carl Zeiss microscope (Madrid, Spain). For the cross-section images, the membrane samples were frozen in liquid nitrogen and fractured. All the samples were gold sputtered to make the samples conductive. The cross-section images of the photocatalytic membranes made it possible to measure the thickness of the different layers. The photocatalyst layer thickness averages values were calculated after measurement in 5 different layer locations.

### 2.3. Hydrogen Production

The photocatalytic hydrogen production experiments were carried out in a 330 mL borosilicate photoreactor using 240 mL of 20% methanol solution as sacrificial agent. The photocatalytic membranes were fixed between two PTFE rings and they were placed in the center of the reactor. The photoreactor was coupled to a gas chromatograph Shimadzu 2010 Plus (Barcelona, Spain) equipped with a Shin Carbon ST 80/100 column using argon as carrier gas, and a thermal conductivity detector. Four Philips PL-S 9W lamps, with a wavelength range between 315 and 400 nm and a maximum emission at 365 nm, were used as light source. The irradiance, measured with a Delta Ohm HD 2102.1 photoradiometer (Padova, Spain), was 7.5 W m^−2^.

The reaction media was bubbled with argon for 30 min in the dark to remove oxygen before the reaction was started. The operation temperature was 20 °C. The experiments were carried out twice to calculate the error bands.

In order to quantify the photocatalyst leaching, the turbidity of the solution was measured with a Turbiquant 3000 IR spectrometer (Merck, Madrid, Spain).

## 3. Results

### 3.1. Materials Characterization

**Figure 2:**
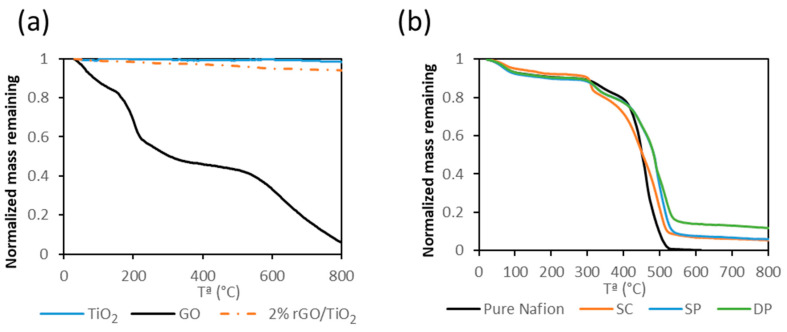
TGA of TiO_2_, pure GO and rGO/TiO_2_ (**a**). TGA of pure Nafion and photocatalytic membranes (**b**).

Figure 2a shows the TGA curves of different solid samples, i.e., TiO_2_, pure GO and rGO/TiO_2_. The GO thermogravimetric curve showed a weight loss up to 100 °C, that corresponded to the water contained in the material. The mass decrease from 150 to 300 °C was attributed to the loss of the oxygen-containing groups. The last mass loss step started around 500 °C and was assigned to the pyrolysis of the GO carbon skeleton [51]. For bare TiO_2_, the initial mass loss was attributed to the adsorbed water in the material. Therefore, comparing the TG curves of the pure compounds and of the rGO/TiO_2_ solid a composition of 3.9% GO in the composite was calculated.

Figure 2b shows the TGA curves of the different membranes. Pure Nafion TGA curve showed three mass loss steps. The first one was attributed to the loss of absorbed water up to 100 °C. The second one, between 300 and 400 °C was related to the decomposition of the –SO_3_H groups. The last step was assigned to the degradation of the polymer backbone between 400 and 550 °C [52,53]. The thermal decomposition of the photocatalytic membranes was similar to that of pure Nafion. However, the decomposition temperature of the polymer backbone was shifted to higher temperatures due to the inorganic content. This fact could be due to the interaction between the Nafion backbone and inorganic particles. TGA results revealed that the content of photocatalyst was 8.8 ± 0.8%, 11.1 ± 3.0%, and 8.9 ± 7.0% in SC, SP, and DP membranes respectively. The variability of the photocatalyst concentration in the DP membrane was attributed to the heterogeneity of the particle concentration distribution on the membrane surface. This fact was due to the difficulty of obtaining a uniform photocatalyst layer on the membrane through this method.

**Figure 3:**
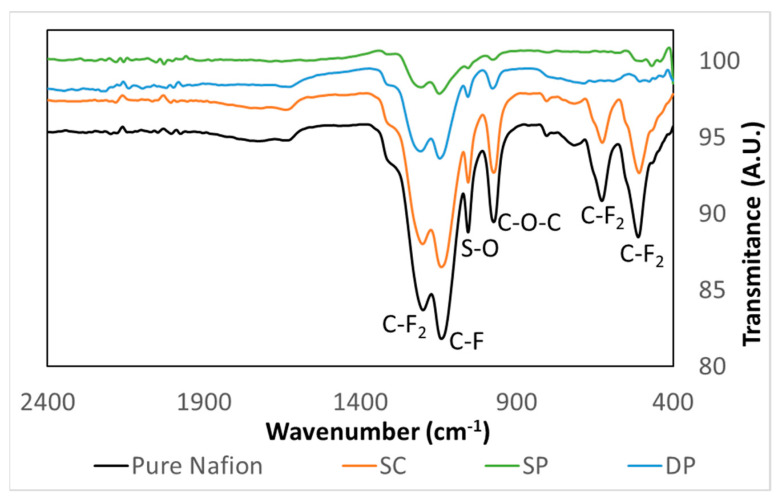
Pure Nafion and photocatalytic membrane FTIR spectra.

**Figure 4:**
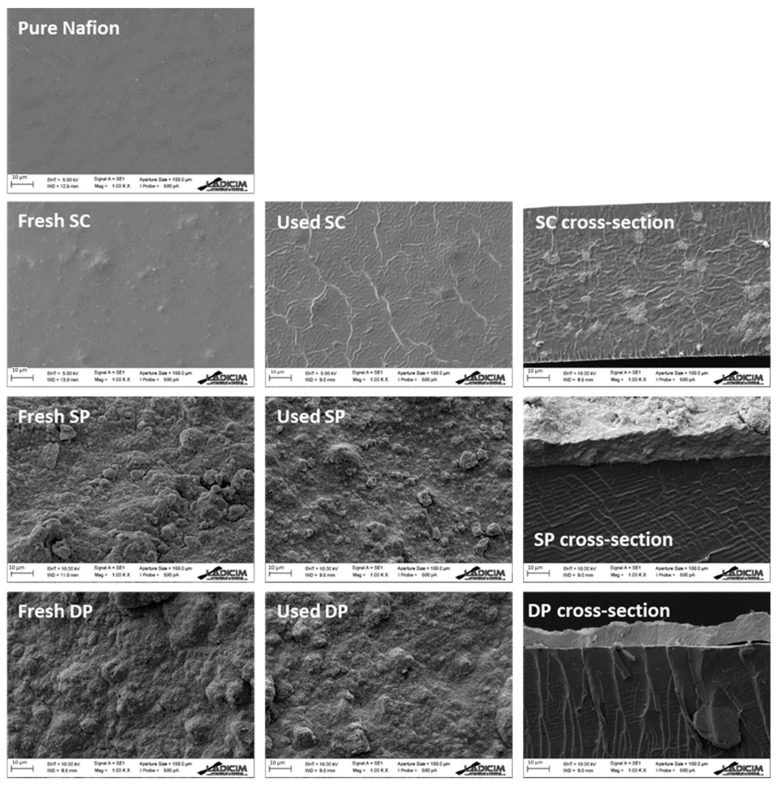
SEM images of pure Nafion, fresh membrane and used membrane surfaces, and fresh membrane cross-section. Scale bar = 10 μm.

Figure 3 shows FTIR spectra of pure Nafion and photocatalytic membranes. The pure Nafion curve showed the typical bands of this material. The bands at 1200 and 1140 cm^−1^ were assigned to the stretching vibrations from C–F_2_ and C–F, respectively. The 1056 and 975 cm^−1^ bands were attributed to S–O stretching vibrations and C–O–C stretching vibrations, respectively. The bands at 627 and 512 cm^−1^ were assigned to C–F_2_ bending vibrations. Photocatalytic membrane spectra showed the typical bands of Nafion suggesting that the addition of photocatalyst does not affect the molecular structure of the Nafion membrane. This fact was also observed by Ding et al. [45]. However, the intensity of these bands was different for the different membranes. Pure Nafion showed the highest band intensity followed by the SC membrane in which most of the photocatalyst is contained inside the membrane matrix. SP and DP membranes showed less intensity in the FTIR spectra because the photocatalyst remains on the surface of the Nafion membranes. The slightly smaller thickness of the photocatalyst layer (9.8 and 12.6 µm in DP and SP membranes, respectively) of DP membranes that was revealed in cross section SEM images (Figure 4) could explain the higher FTIR signal intensity in the DP compared to the SP membrane.

Figure 4 shows the SEM surface images of the fresh and used membranes, and the cross section of the fresh membranes. The fresh SC SEM image revealed bulges in the membrane surface, which indicated the presence of photocatalyst inside the membrane matrix. Meanwhile, the micrographs of the fresh SP and DP membranes revealed that photocatalyst covered the whole Nafion surface.

The comparison between fresh and used SC membranes showed a ribbed surface in the used SC membrane indicating damage in the Nafion structure during the hydrogen production process. The rest of the used membranes did not show any appreciable change on the surface after the hydrogen production process.

The SC membrane cross-section revealed the presence of the photocatalyst in the whole membrane matrix, whereas in the SP and DP membrane cross-sections the expected photocatalyst layer deposition on the surface of the Nafion membrane was observed; photocatalyst layer thickness and membrane thickness were also determined (Table 1).

### 3.2. Photocatalytic Membrane Performance

**Figure 5:**
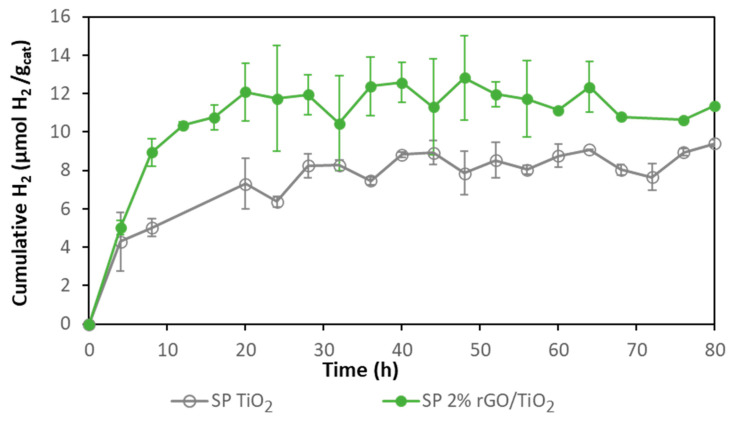
Comparison of hydrogen production with 2% rGO/TiO_2_ SP and bare TiO_2_ SP membranes.

In order to confirm the higher photocatalytic activity of the composite photocatalyst compared with bare TiO_2_ when they are immobilized on Nafion membranes, SP and TiO_2_ immobilized catalysts were tested for photocatalytic hydrogen production (Figure 5). Although the initial rate of hydrogen production with both photocatalysts was similar (ca. 1.1 µmol H_2_ gcat^−1^ h^−1^), the initial rate using TiO_2_ decreased after 4 h and reached a total hydrogen production of 9 µmol H_2_ gcat^−1^ while the composite decreased its initial rate after 8 h with a total hydrogen production of 12 µmol H_2_ gcat^−1^. It was confirmed that 2% rGO/TiO_2_ photocatalytic membranes performed better than TiO_2_ membranes for hydrogen production.

**Figure 6:**
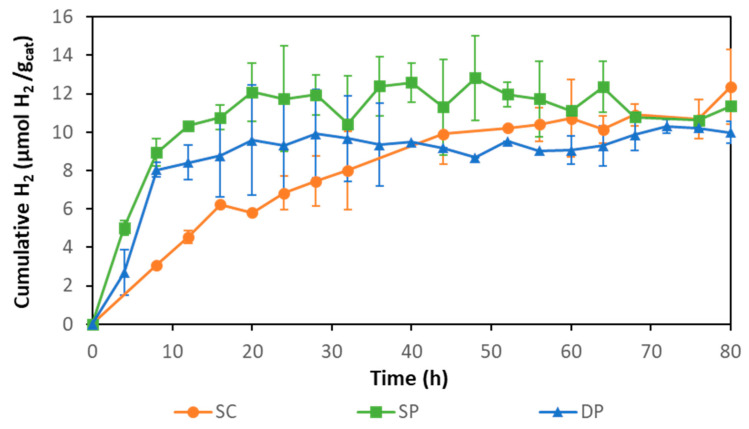
Hydrogen production with SC, SP, and DP photocatalytic membranes.

Membranes obtained following the three different photocatalyst immobilization methods were tested for hydrogen production (Figure 6). Composite SP and DP membranes achieved HPRs of 1.11 ± 0.09 and 1.01 ± 0.10 µmol H_2_ gcatalyst^−1^ h^−1^, respectively. With these membranes, hydrogen production was stopped after around 20 h. The composite SC membrane achieved an initial rate of 0.38 ± 0.03 µmol H_2_ gcatalyst^−1^ h^−1^ during the first 8 h, but the hydrogen production continued to increase smoothly and stopped around 60 h of photoreaction. The higher initial activity of SP and DP membranes was attributed to the higher accessibility to the photocatalyst provided by these membranes, as the catalyst was mainly deposited on the membrane surface while SC membranes had most of the photocatalyst embedded in the membrane matrix, thus, exerting higher resistance to mass transport of the sacrificial agent.

The three types of photocatalytic membranes reached a total hydrogen production ca. 11 µmol H_2_ gcat^−1^ after 80 h. Hydrogen production stopped most likely due to the inhibitory effect of accumulated hydrogen in the system as has been previously reported [48,54]. The accumulated hydrogen could reduce the difference between the H^+^ reduction potential and the TiO_2_ conduction band potential, diminishing the driving force for hydrogen production until it finally stops.

In our previous work, 2% rGO/TiO_2_ composite suspensions were used for photocatalytic hydrogen production in the same experimental setup with the same equivalent photocatalyst concentration (0.18 g/L), achieving an initial HPR of 1.6 µmol H_2_ gcatalyst^−1^ h^−1^ [55]. Comparing this value with the highest hydrogen production initial rate achieved by the immobilized photocatalyst (1.1 µmol H_2_ gcatalyst^−1^ h^−1^), a reduction of 30% is deduced when the immobilized photocatalyst is used. Filice et al. observed different behavior in the degradation of methyl orange [43]. These authors reported that the photocatalyst immobilized on Nafion membranes achieved higher pollutant removal than in suspension. Vohra and Tanaka observed an improvement in the photocatalytic activity in Paraquat degradation by coating Nafion with TiO_2_ [46]. In this work, the decrease in HPR when using the IMPR vs the slurry reactor is explained by the increase in the mass transfer resistance that the sacrificial agent must overcome before reaching the catalyst.

Next, the catalyst leaching from the membranes was evaluated; to this end the turbidity of the solution before and after the photocatalytic process was analyzed. A linear relationship was found between turbidity and the composite concentration in suspension in the range between 0 and 10% photocatalyst (*w*/*w*). Therefore, knowing the turbidity in the solution the amount of photocatalyst in suspension that was leached from the membrane was determined. Table 2 shows the solution turbidity and the resulting percentage of photocatalyst leached after 80 h of reaction. SC and SP showed the lowest percentage of photocatalyst leaching. DP showed the highest percentage of photocatalyst leaching with a more than ten-fold increase compared to the other methods. SC showed an extremely low leaching percentage because the photocatalyst was embedded in the membrane matrix, thus showing higher stability. The high leaching percentage of DP membranes could be because the photocatalyst was weakly deposited on the membrane surface. Although SP photocatalyst was also deposited on the membrane surface, it resulted in a lower leaching percentage than DP, very similar to SC membranes. This fact could be attributed to a stronger photocatalyst attachment on the membrane surface by the SP method than by the DP method.

The highest hydrogen production initial rate together with the low photocatalyst leaching provide preliminary information for decision making for the catalyst immobilization procedure; thus, the SP membrane turned out to be the best method to immobilize the composite on a Nafion membrane.

**Figure 7:**
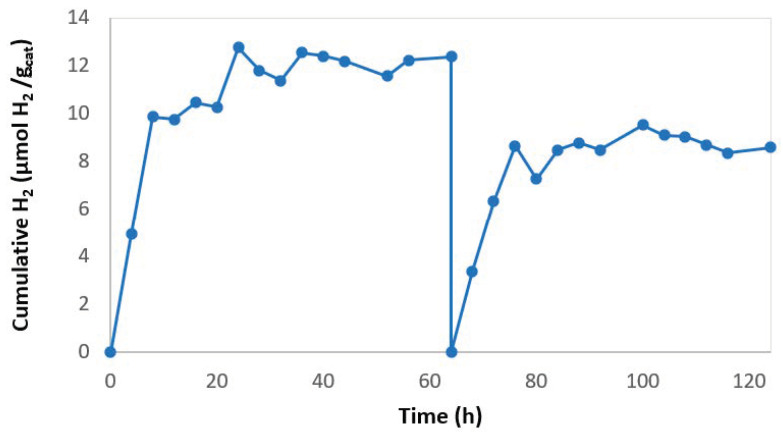
Hydrogen production with reused 2% rGO/TiO_2_ SP membrane.

In order to evaluate the reuse of the SP membrane several cycles of hydrogen production were carried out. After each cycle, the system was purged with argon to prevent the inhibitory effect of the accumulated hydrogen (Figure 7). It was observed that at the end of the first cycle hydrogen production had stopped and after an argon purge, hydrogen production continued. This fact supports the inhibitory effect of the reaction product on the HPR, as was expected. The initial rate in the reuse cycles was 0.79 µmol H_2_ gcatalyst^−1^ h^−1^ while with the fresh membrane it was 1.19 µmol H_2_ gcatalyst^−1^ h^−1^. Therefore, the membrane photoactivity had been reduced by 33%. Ma et al. evaluated the re-use of Er^3+^:YAlO_3_Pt-TiO_2_ on a glass substrate [32]. They carried out 5 cycles of 5 h. The loss of photocatalyst activity after 25 h was 80%, while in this work the loss of activity after 64 h of operation was quantified as 33%.

**Figure 8:**
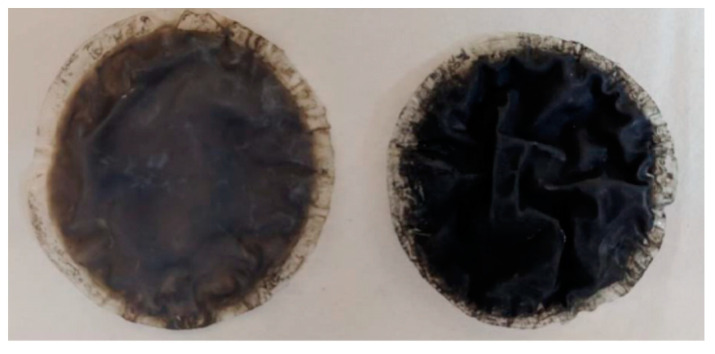
2% rGO/TiO_2_ membrane before (**left**) and after 96 h of use (**right**).

Darkening was observed in the used membranes in comparison with the pristine photocatalytic membranes (Figure 8). This darkening could be attributed to the further reduction of graphene. The reduction of graphene during photocatalytic experiments has been previously reported. Shah et al. observed darkening after reduction of graphene oxide [56]. In our previous study, darkening of TiO_2_/rGO composites was observed after being used in photocatalytic hydrogen production in slurry photoreactors, the reduction of the composite being confirmed through Raman spectra [55]. A decrease in the photoactivity after the first hydrogen production cycle was also observed.

## 4. Conclusions

Although there are environmental and energy advantages of photocatalytic hydrogen production, its large-scale application is still far from adoption, mainly due to the low performance and short lifetime of the state of the art photocatalysts. This work takes a step toward by advancing the knowledge on the application of immobilized composite photocatalysts. Cost-effective rGO/TiO_2_ composite was synthesized showing improved photocatalytic properties compared to bare TiO_2_ membranes. In addition, the influence of different process variables such as the composite immobilization method, SC, SP, and DP, to attach the catalyst to Nafion membranes was analyzed. SP and DP achieved the highest initial HPRs, about 1 µmol H_2_ gcatalyst^−1^ h^−1^. However, the SP method showed only 0.4% photocatalyst leaching after 80 h of operation, whereas DP leaching resulted in 4.8% at the same time. This fact could be due to a stronger photocatalyst attachment on the SP membrane surface than on the DP membrane surface. Therefore, SP seems to be the most effective method to immobilize the composite on Nafion membranes.

The higher initial hydrogen generation rate of SP and DP compared to SC membranes was attributed to the easier accessibility to the photocatalyst deposited on the membrane surface in the two former cases, confirmed by SEM analysis; this reduced the mass transfer limitations in the transport of methanol to reach the immobilized catalyst.

An SP composite reused membrane showed 33% decrease in the initial rate in comparison with a fresh membrane after 64 h of operation. This fact may be attributed to the further reduction of GO in the composite during the photocatalytic process.

## Figures and Tables

**Table 1:** Photocatalyst layer and membrane thickness.

Thickness	Solvent-Casting	Spraying	Dip-Coating
Photocatalyst Layer (µm)	n.a.	12.6 ± 2.6	9.8 ± 2.1
Membrane Thickness (µm)	72.0 ± 0.7	165.6 ± 1.6	162.5 ± 1.2

n.a.: not applicable.

**Table 2:** Leaching percentage of the immobilized photocatalytic membranes.

Parameter	SC	SP	DP
Turbidity (NTU)	2.6 ± 0.9	7.5 ± 2.5	64.3 ± 12.9
Photocatalyst Leaching (%)	0.2 ± 0.1	0.4 ± 0.2	4.8 ± 1.0
